# Supplementary material for: LEAFY is a pioneer transcription factor and licenses cell reprogramming to floral fate
Source: Nat Commun. 2021 Jan 27;12:626. doi: 10.1038/s41467-020-20883-w (PMC7840934; doi:10.1038/s41467-020-20883-w)
Supplement: Supplementary file 1 — Supplementary Information [file 41467_2020_20883_MOESM1_ESM.pdf]

LEAFY is a pioneer transcription factor and licenses cell reprogramming to floral fate

Supplementary Information

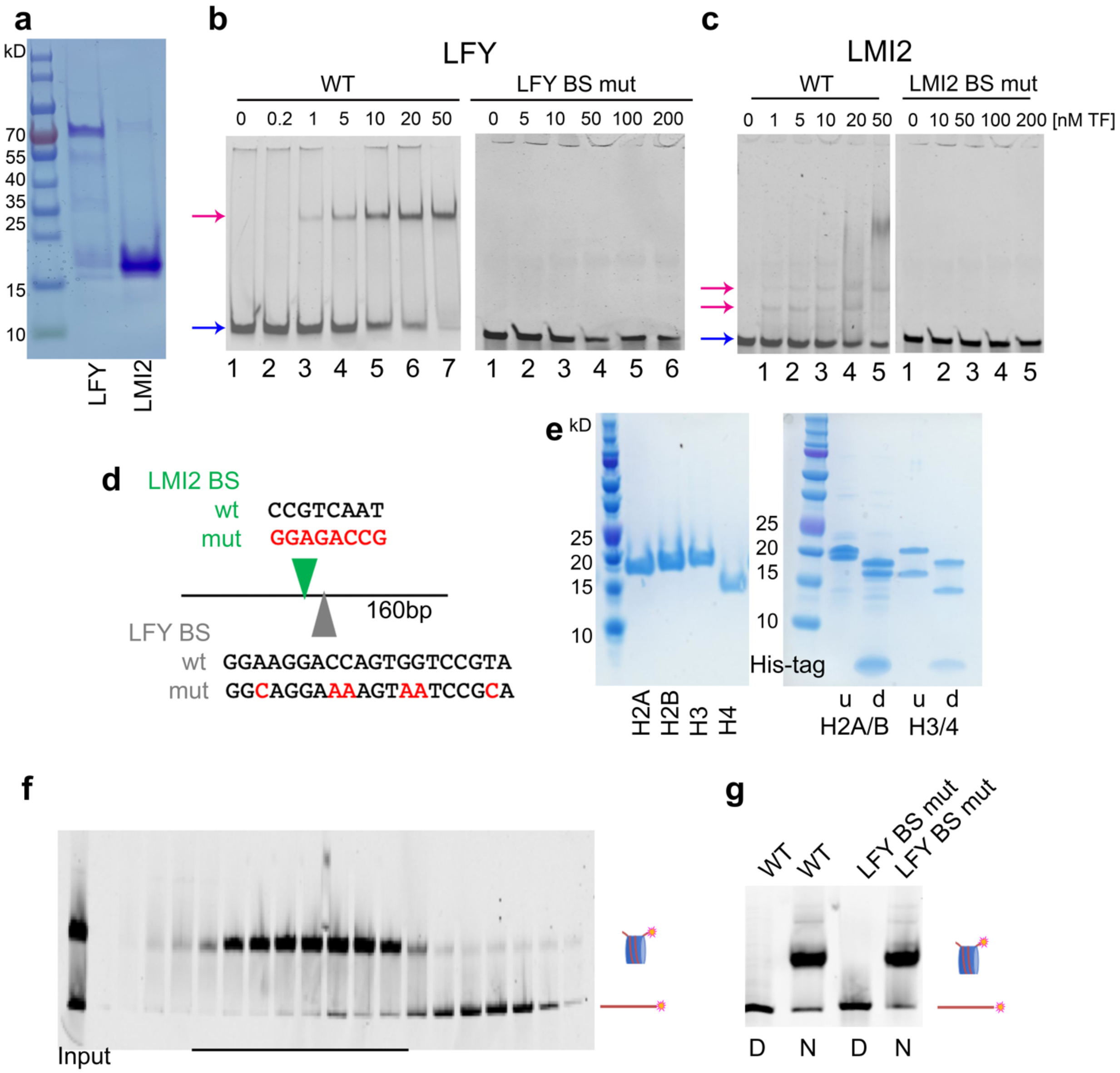

Supplementary Figure 1. Nucleosome assembly and Electrophoretic Mobility Shift Assays

a, LMI2 and LFY proteins purified by Ni-NTA and anion exchange chromatography as described in the methods. Proteins were of expected sizes and desired purity. Left: sizes (in kD). The experiment was repeated two times with similar results. **b, c,** EMSA showing LFY (b) or LMI2 (c) binding to the naked Cy5-labeled 160 base-pair

endogenous *AP1* regulatory region DNA. Right: *AP1* regulatory region with wild-type binding motif (WT). Left *AP1* regulatory region with mutated binding motif (BS mut). As there is only one LMI2 binding site in the 160 bp probe, the multiple bands observed in the LMI2 gel shift with the *AP1* regulatory region (c, left) are likely attributable to LMI2 homodimers and higher order multimers. The experiment was repeated three times with similar results. Neither LFY nor LMI2 bound the mutated DNA at molar excess. See panel (d) for motif changes. Arrows point to unbound DNA (blue) and bound, shifted DNA (red). **d,** Native *AP1* regulatory region DNA (TAIR10 Chr1:25,986,456 – 25,986,608) containing wild-type or mutated LFY and LMI2 binding sites. The entire LMI2 site was altered since this motif has not yet been functionally dissected. **e,** Left: Core histones after purification. Sizes (in kD). Right: H2A/B and H3/4 dimer assembly and His-tag removal by thrombin digestion. u = undigested, d = digested. The lower bands in the digested lanes are cleaved His-tags. The experiment was repeated two times with similar results. **f,** Glycerol gradient purification of nucleosomes assembled with Cy5-labeled native *AP1* DNA. Black lines: gradient fractions used for further analyses. **g,** Assembled nucleosomes (N) after glycerol gradient as well as free DNA (D). Symbols: labelled DNA (red line), nucleosome (red lines and histones) (f, g).

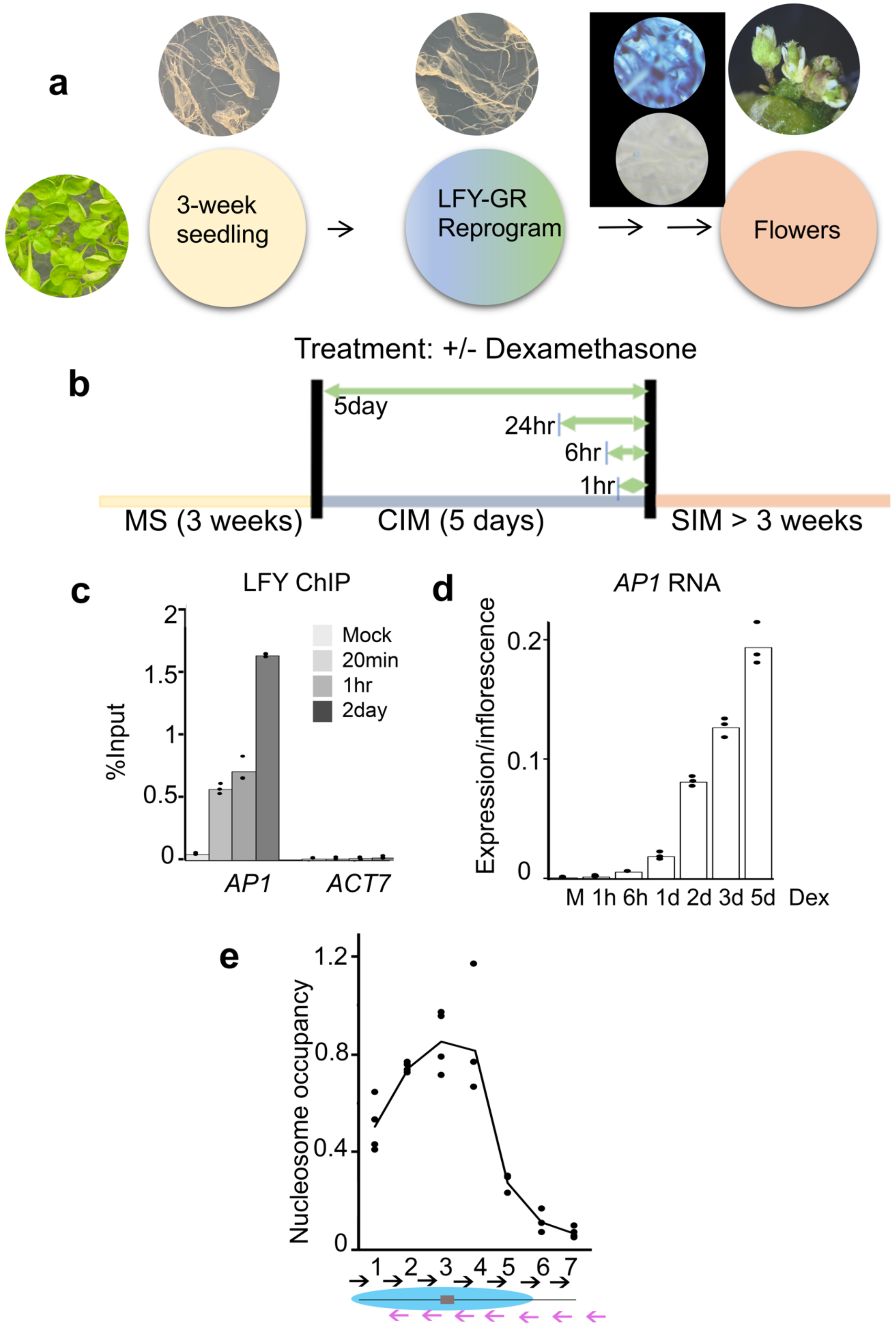
a
3-week seedling
LFY-GR Reprogram
Flowers
Treatment: +/- Dexamethasone
b
5day
24hr
6hr
1hr
MS (3 weeks)
CIM (5 days)
SIM > 3 weeks
c
LFY ChIP
%Input
2
1.5
1
0.5
0
Mock
20min
1hr
2day
AP1
ACT7
d
AP1 RNA
Expression/inflorescence
0.2
0.1
0
M 1h 6h 1d 2d 3d 5d Dex
e
Nucleosome occupancy
1.2
0.8
0.4
0
1 2 3 4 5 6 7

Supplementary Figure 2. Root explant reprogramming to floral fate.
a, Reprogramming of root explants by 35S:LFY-GR to flower fate. For reprogramming, roots were treated with the dexamethasone steroid and transferred to shoot inducing medium as previously described [1]. Black box: Staining of *pAP1*:GUS [1] in root explants eight days after dexamethasone (top) or mock treatment (bottom). **b,** Roots of 35S:LFY-GR seedlings grown on MS plates were incubated on callus inducing medium (CIM) for 5 days. During this time, plants were treated between 1 hour and 5 days with the synthetic steroid dexamethasone (dex; 5 μM in 0.1% ethanol) or mock (0.1 % ethanol) solution. All treatments terminated at ZT6 at the end of the 5-day CIM incubation. **c,** To assess the kinetics of LFY recruitment to the *AP1* locus, root explants were treated for indicated time periods prior to ZT6 at day 5 with dexamethasone or mock solution. Strong LFY occupancy at the locus was observed 20 minutes after LFY activation. **d,** To determine the kinetics of *AP1* upregulation, root explants on CIM were treated for indicated time periods prior to ZT6 at day 5 with dexamethasone or mock solution. Upregulation of *AP1* was first detected 24 hours after LFY-GR activation and increased further until day five. Expression is shown relative to that of six-week old inflorescence apices with associated closed flower buds and flower primordia, which denotes maximal *AP1* expression (ca. three times the *AP1* level of plants transitioning to floral fate [2]). M: mock treatment. **e,** MNase-qPCR was performed at day 5 on CIM in root explants with a tiled primer array [3] flanking the LFY motif [4-6]. This uncovered a well-positioned nucleosome with the nucleosome center (dyad) in the middle of the primer pair 3 amplicon, at the LFY binding site. The signal for each primer pair was assigned to the midpoint of the 80 bp PCR product. Below: tiling primer pairs 1 to 7, offset by 40 bp. Nucleosome (blue ellipse) with LFY binding site (grey rectangle) at the midpoint of primer pair 3. **c-e**, Black dots: individual data points for technical replicates, (n=1). Experiments were repeated two times with similar results.

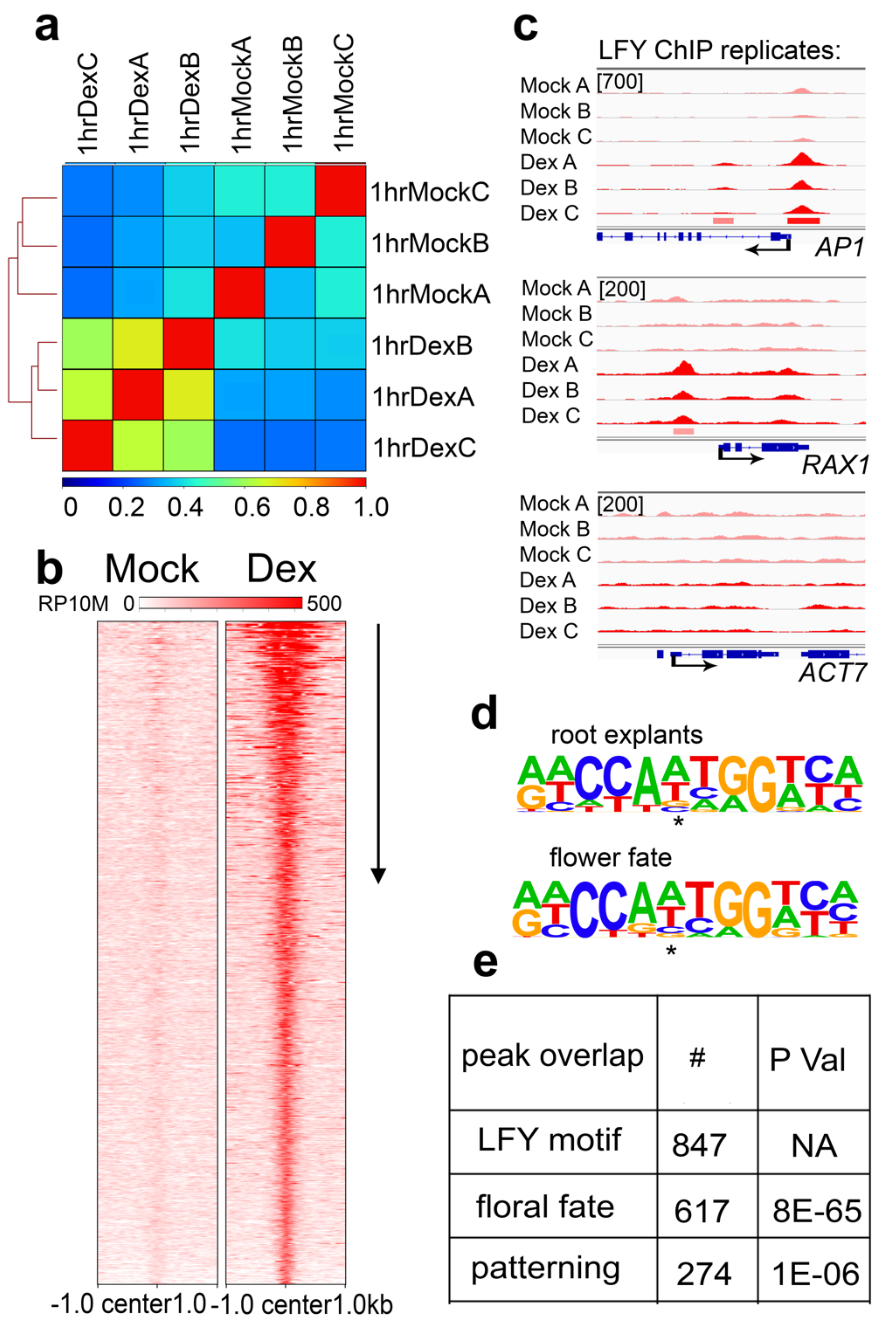

peak overlap	#	P Val
LFY motif	847	NA
floral fate	617	8E-65
patterning	274	1E-06

Supplementary Figure 3. LFY ChIP-seq data quality
a, Spearman correlation coefficients of one-hour mock (LFY cytoplasmic) or dexamethasone steroid (LFY nuclear) treated 35S:LFY-GR ChIP-seq replicates from root explants. Correlation analysis was conducted for significant LFY peak-regions (MACS2 summit *q* value ≤ 10^{-10}). **b,** Heatmap of 35S:LFY-GR ChIP-seq binding peaks after mock or dexamethasone treatment centered on dexamethasone treated LFY peak summits and ranked from lowest to highest summit *q* value (arrow). Legend: RP10M. **c,**

Browser view of mock and dexamethasone treated 35S:LFY-GR ChIP-seq replicates at the *AP1, RAX1* and *ACT7* loci. Significant ChIP peaks (summit q-value≤10^{-10} MACS2 of pooled replicates) are marked by horizontal bars, with the color saturation proportional to the negative log 10 *q* value (as for the narrowPeak file format in ENCODE). **d,** Most highly ranked *de novo* motif (HOMER [7]) under significant LFY peak summits in root explants (top, *p* value 1E-86; this data) or during the switch to flower fate (bottom; *p* value 1E-145; Ref. [8]). Asterisk: center of the palindrome. **e,** Overlap of identified LFY peaks (n=1177) with known LFY motif [5,6,8], with LFY peaks during the transition to floral fate in seedlings (n=1298) [6], or during flower patterning in the inflorescence (n=748) [6]. *p* values based on hypergeometric test assuming n=4000 possible binding events. NA: not applicable.

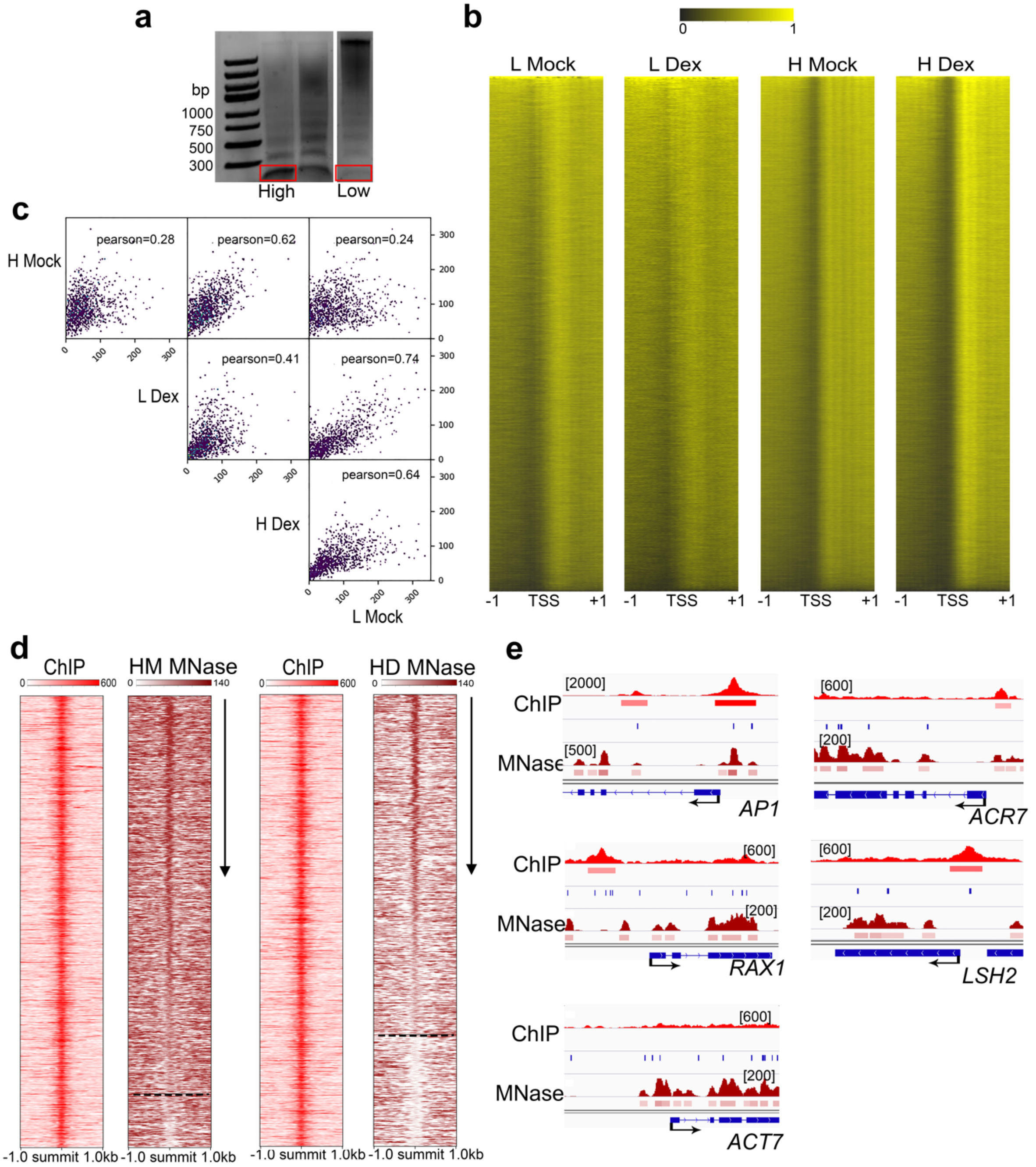

Supplementary Figure 4. LFY MNase-seq data quality

a, Isolation of mono-nucleosomes (red box) after standard (high; 0.5 units MNase/µl, left) or low (0.05 units MNase/µl, right) digestion. The experiment was repeated two times with similar results. **b,** Total MNase-seq signal within ± 1kb of the transcription start sites (TSS). 35S:LFY-GR root explants were treated for one hour with mock solution followed by low MNase digestion (L Mock) or high MNase digestion (H Dex) or treated for one hour with dexamethasone followed by low MNase digestion (L Dex) or high MNase digestion (H Dex). The expected nucleosome phasing over gene bodies [9]

is observed as is loss of fragile nucleosomes immediately upstream of the transcription start[10] site in the high digestion. Legend above: signal intensity. **c,** Pearson correlation coefficient comparison of the MNase datasets generated. **d,** Significant LFY ChIP-seq signal ((RP10M; MACS2 q-value≤10^{-10}) and MNase-seq nucleosome occupancy (DANPOS, *q* value≤10^{-50}) in the 2 kb region centered on the LFY peak summits. Heatmaps were ranked by nucleosome occupancy (arrow) in the LFY peak summit region (± 75 bp). Samples were subjected to high MNase digestions after mock (M) or steroid treatment (D) of LFY-GR. Dotted line separates nucleosome occupied (top) from nucleosome free (bottom) LFY binding sites. **e,** Browser view of *AP1*, *RAX1*, *ACT7*, *LSH2* and *ACR7* loci showing LFY ChIP-seq (light red), MNase-seq signal (Mock, dark red) and LFY binding motifs (blue). Signals for ChIP peaks (summit *q* value ≤ 10^{-10} MACS2) or nucleosome occupancy (*q* value ≤ 10^{-50}, DANPOS) are marked by horizontal bars, with the color saturation proportional to the signal strength.

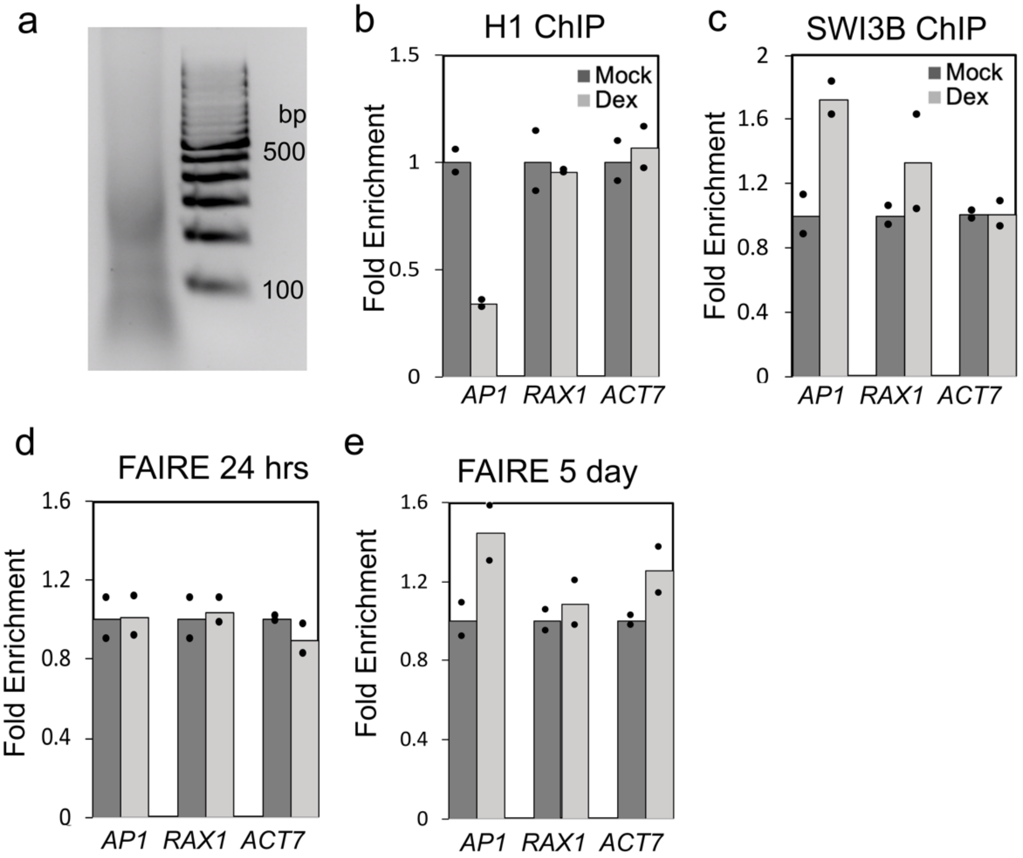

Supplementary Figure 5. Independent replicates for chromatin opening and accessibility

a, High chromatin sonication for sequential ChIP. Right: sizes (in bp). The experiment was repeated two times with similar results. **b-e** Independent ChIP and FAIRE experiments. Root explants were treated for 24 hours with mock or dexamethasone (Dex) solution unless indicated otherwise. Fold enrichment is shown relative to the mean of mock treated samples for each locus. Black dots: means from n = 2 independent biological experiments. **b**, H1 ChIP-qPCR at the LFY bound sites. **c,** SWI/SNF complex subunit SWI3B ChIP-qPCR at LFY bound sites. **d, e,** FAIRE qPCR at known DNase hypersensitive sites [11] twenty-four hours after LFY activation (d) or five-days after LFY activation (e).

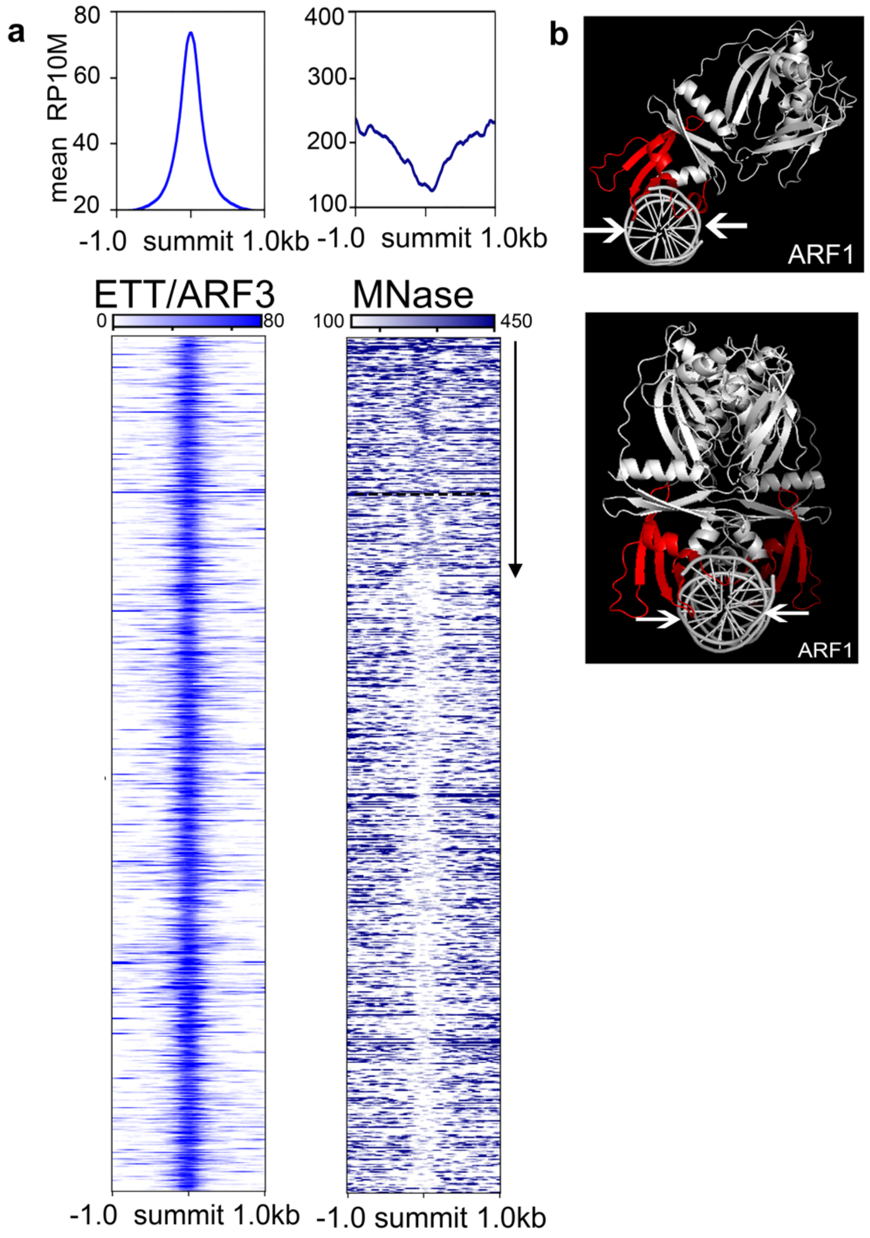

Supplementary Figure 6. ETT preferentially binds naked DNA

a, Top: Mean inflorescence ETT/ARF3 [12] ChIP-seq signal (RP10M, q value $\leq 10^{-10}$, MACS2) and nucleosome [13] occupancy signal (DANPOS q value 10^{-300}) in the region ± 1 kb of significant ETT/ARF3 peak summits. Bottom: heatmap of inflorescence [12,13] ETT/ARF3 ChIP-seq and MNase-seq signal (RP10M) in a 2 kb region centered on significant ETT/ARF3 peak summits sorted on nucleosome occupancy (arrow) in ETT/ARF3 peak summit regions (±75bp). Dotted line separates nucleosome occupied (top) from nucleosome free (bottom) ETT/ARF3 binding sites. **b,** Structure of the DNA-anchoring beta sheets and loops of ARF1 (PDB: 4LDX; DNA bound ARF1 DBD

[https://www.rcsb.org/structure/4DLX]), which is closely related to ETTIN/ARF3[14]. Arrows delineate the DNA region contacted. No structure is available for ARF3.

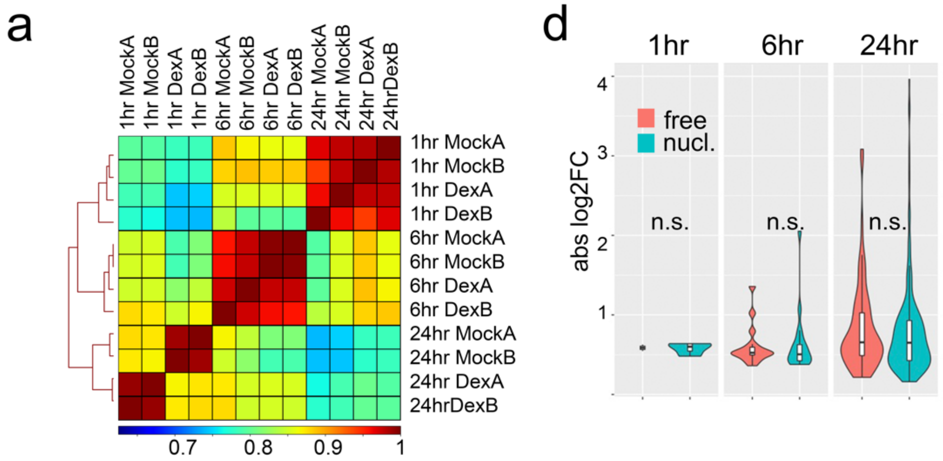

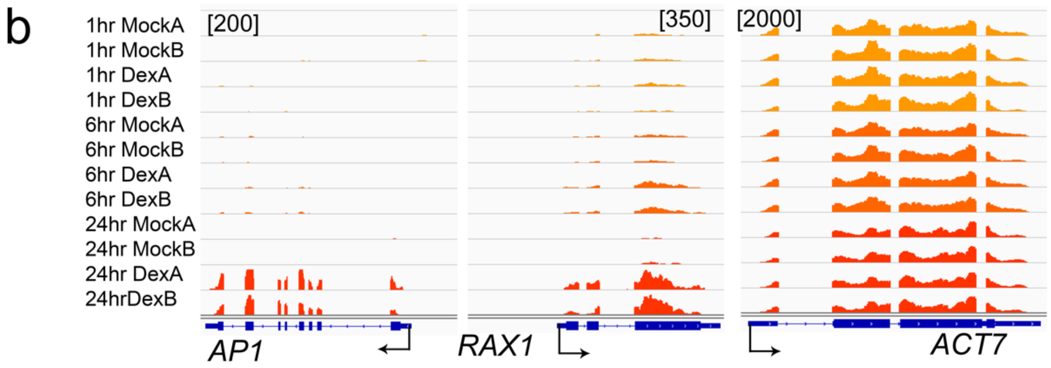

c

Category \ Time Point	1hr Dex	6hr Dex	24hr Dex
Known LFY targets	3	21	133
Total	5	33	302
Hyperg. pval	0.5	1E-07	1E-21

Supplementary Figure 7. Transcriptional changes and chromatin state after LFY-GR activation in root explants.

a, Spearman correlation heatmap of RNA-seq reads from root explants mock or steroid treated for different durations. A clear separation of the LFY activated transcriptome is observed twenty-four hours after steroid treatment. **b,** Browser view of *AP1*, *RAX1* and *ACT7* loci showing RNA-seq reads in two replicates for each timepoint and treatment condition. **c,** Comparison of LFY bound and regulated genes from root explants with known LFY targets (n=2933) [6]. *p* value: Hypergeometric test relative to all expressed genes (n=15,000). **d,** Absolute expression fold change of all significantly differentially expressed genes (DESeq2, *q* value < 0.01 steroid versus mock treatment). Red: LFY target genes associated with a LFY bound site that was nucleosome occupied (nucl.).

Turquoise: LFY target genes associated with a LFY bound site in free DNA (free). n (from left to right) = 1, 4, 12, 21, 101, 201. n.s. = 0.92. 0.92, 0.51 for one, six and twenty-four hours; two-tailed students' t-test. Violin plots depicting mean (horizontal black line), interquartile range (white box), 95% confidence value (vertical black line) and frequency (density plot width).

Supplementary Table 1.

LOCI probed	Primers for Seq. ChIP & LFY ChIP qPCR	Primers for FAIREqPCR:	Primers for SWI3B & H1 ChIP qPCR:
AP1 F	Same as MNase primer set 3	TGGCCTTGAAGACAA CATCC	Same as Seq.ChIP primers
AP1 R	Same as MNase primer set 3	TGGGAAGAAAAGAA GGGTGGAG	Same as Seq.ChIP primers
RAX1 F	GTCCGTCCAATGCAA TCTTC	Same as Seq.ChIP primers	Same as Seq.ChIP primers
RAX1 R	AACAGTCCCAAGTGG TGTTG	Same as Seq.ChIP primers	Same as Seq.ChIP primers
ACTIN7 F	CGTTTCGCTTTCCTT AGTGTTAGCT	Same as Seq.ChIP primers	Same as Seq.ChIP primers
ACTIN7 R	AGCGAACGGATCTAG AGACTCACCTTG	Same as Seq.ChIP primers	Same as Seq.ChIP primers
LSH2 F	TGG GTC GTC TGA AGA AAG AG		
LSH2 R	AGT TTG TGT CCG GAA TCA CG		
ACR7 F	TTTGAACAATAATGC TGCCTTC		
ACR7 R	TGGACAAAATTGAAG TGGTTTAG		
Primers for Gibson Assembly:			
LMI2 BS mut F	GAAATCTCCGGGAGACCGGCAAAGCGAAGGTGACACTTG		
LMI2 BS mut R	GCCGGTCTCCCGGAGATTTCCCTGTAGATCTACGAAAC		
LFY CDS into pE-SUMO F	GTGAAACCTTCAGGATCCATACCTCCAATCTGTTCGCGGTGAGCCTCAATA ATATCGTTA		
LFY CDS into pE-SUMO R	GCGACGACTTGCGTTTCTAGACTAGAGGATCCGAATTCGAGCTCCGTCGA CAAGCTTGC		
LMI2 CDS into pE-SUMO F	CAACAAGGTGTTCTTCCCATACCTCCAATCTGTTCGCGGTGAGCCTCAATA ATATCGTTA		
LMI2 CDS into pE-SUMO R	CCATGGTTTCCAAATTCTAGACTAGAGGATCC GAATTCGAGCTCCGTCGACAAGCTTGC		
Primers for pENTR-dTOPO cloning of LMI2-3HA:			
LMI2_3HA_F	CACCATGGGAAGAACACCTTGT		
LMI2_3HA_R	GCTGCACTGAGCAGCG		
Primers for qRT-PCR:			
AP1 F	GCAAGCAATGAGCCCTAAAGAG		
AP1 R	AGTGCGGATGTGCTTAAGAGC		
Primers for MNase qPCR:			
AP1 1F	ACTAATGTCGGGTCCATGAT		
AP1 1R	CGAGACGTCGATAATCAAATTGT		
AP1 2F	GCGGACTTAAAAATATGAAAATAAACAATTTG		

AP1 2R	CCAGTGGTCCGTACAATG	
AP1 3F	CGTGAAGAGAAATGGGTAAGTA	
AP1 3R	CGTCAATGCAAAGCGAAG	
AP1 4F	TCCTTCCCCAAGTGTCAC	
AP1 4R	GGTTCAGATTTTGTTTCGTAGATC	
AP1 5F	CGGAGATTTCCCTGTAGATC	
AP1 5R	AGAGAAATGTTAATAAAAGGAAATTAAAAATAGAT	
AP1 6F	CTGAACCAACCAAAATATCTATTTTTAATTT	
AP1 6R	CTTATTCCAAAAGAATAGTGTAAAATAGGG	
AP1 7F	CCTTTTATTAACATTTCTCTATTACCCTATTT	
AP1 7R	AAGCAAATTTGATAAAACAAAGGGTT	

Primers for pENTR-3C cloning OF SWI3B:

3B Kpn5	ATAGGTACCACGTTCCAATTTCTACCTACTT	For amplifying 311bp upstream and whole genomic SWI3B
3B Eag3	CATCGGCCGACACTCTATTCTATCTTCAG	-“-
HA-F-EagI	AACCGGCCGGGGTTAATTAACATCTTTTACCC	For amplifying 3xHA for fusion to 3'UTR SWI3B
HA-R-XhoI	TTACTCGAGGCTGCACTGAGCAGCGTAATCTG	
3B 3UTR Xho5	AGCTCGAGTAGAACGCACTTAATTTGAAAC	For amplifying 3'UTR SWI3B
3B 3UTR Xba3	TATTCTAGAGCCATTTGGTTTTGACTTTTA	-“-

Supplemental References

1. Wagner, D. *et al.* Floral induction in tissue culture: a system for the analysis of LEAFY-dependent gene regulation. *Plant J* **39**, 273-82 (2004).
2. Yamaguchi, A. *et al.* The microRNA-regulated SBP-Box transcription factor SPL3 is a direct upstream activator of LEAFY, FRUITFULL, and APETALA1. *Dev Cell* **17**, 268-78 (2009).
3. Han, S.K. *et al.* The SWI2/SNF2 chromatin remodeling ATPase BRAHMA represses abscisic acid responses in the absence of the stress stimulus in Arabidopsis. *Plant Cell* **24**, 4892-906 (2012).
4. Benlloch, R. *et al.* Integrating long-day flowering signals: a LEAFY binding site is essential for proper photoperiodic activation of APETALA1. *Plant J* **67**, 1094-102 (2011).
5. Moyroud, E. *et al.* Prediction of regulatory interactions from genome sequences using a biophysical model for the Arabidopsis LEAFY transcription factor. *Plant Cell* **23**, 1293-306 (2011).
6. Winter, C.M. *et al.* LEAFY Target Genes Reveal Floral Regulatory Logic, cis Motifs, and a Link to Biotic Stimulus Response. *Dev Cell* **20**, 430-43 (2011).
7. Heinz, S. *et al.* Simple Combinations of Lineage-Determining Transcription Factors Prime cis-Regulatory Elements Required for Macrophage and B Cell Identities. *Molecular Cell* **38**, 576-589 (2010).
8. Sayou, C. *et al.* A SAM oligomerization domain shapes the genomic binding landscape of the LEAFY transcription factor. *Nat Commun* **7**, 11222 (2016).
9. Soufi, A. *et al.* Pioneer transcription factors target partial DNA motifs on nucleosomes to initiate reprogramming. *Cell* **161**, 555-568 (2015).
10. Iwafuchi-Doi, M. *et al.* The Pioneer Transcription Factor FoxA Maintains an Accessible Nucleosome Configuration at Enhancers for Tissue-Specific Gene Activation. *Mol Cell* **62**, 79-91 (2016).
11. Zhang, W., Zhang, T., Wu, Y. & Jiang, J. Genome-wide identification of regulatory DNA elements and protein-binding footprints using signatures of open chromatin in Arabidopsis. *Plant Cell* **24**, 2719-31 (2012).
12. Simonini, S., Bencivenga, S., Trick, M. & Ostergaard, L. Auxin-Induced Modulation of ETTIN Activity Orchestrates Gene Expression in Arabidopsis. *Plant Cell* **29**, 1864-1882 (2017).
13. Choi, K. *et al.* Nucleosomes and DNA methylation shape meiotic DSB frequency in Arabidopsis thaliana transposons and gene regulatory regions. *Genome Res* **28**, 532-546 (2018).
14. Mutte, S.K. *et al.* Origin and evolution of the nuclear auxin response system. *Elife* **7**(2018).
