# Supplementary material for: LEAFY is a pioneer transcription factor and licenses cell reprogramming to floral fate
Source: Nat Commun. 2021 Jan 27;12:626. doi: 10.1038/s41467-020-20883-w (PMC7840934; doi:10.1038/s41467-020-20883-w)
Supplement: Supplementary file 3 — Descriptions of Additional Supplementary Files [file 41467_2020_20883_MOESM3_ESM.pdf]

Descriptions of Additional Supplementary Files

Supplementary data 1

Description: Summary of genomic datasets generated in this study. a. LFY binding peak overlap with a nucleosomes in all four MNase-seq datasets generated. b. Genes annotated to significant LFY binding peaks. c. Genes significantly differentially expressed (DeSeq2) after steroid activation of LFY-GR relative to mock treatment. Treatment durations were one, six or twenty-four hours.

Supplementary data 2

Description: Gene ontology terms enrichment analysis. a. GO terms for LFY targets where the LFY binding peak was nucleosome occupied. b. GO terms for LFY targets where the LFY binding peak was a in nucleosome free genomic region.
