# Supplementary material for: LEAFY is a pioneer transcription factor and licenses cell reprogramming to floral fate
Source: Nat Commun. 2021 Jan 27;12:626. doi: 10.1038/s41467-020-20883-w (PMC7840934; doi:10.1038/s41467-020-20883-w)
Supplement: Supplementary file 6 — Reporting Summary [file 41467_2020_20883_MOESM6_ESM.pdf]

nature research

Corresponding author(s): Doris Wagner

Last updated by author(s): 12/05/2020

Reporting Summary

Nature Research wishes to improve the reproducibility of the work that we publish. This form provides structure for consistency and transparency in reporting. For further information on Nature Research policies, see Authors & Referees and the Editorial Policy Checklist .

Statistics

For all statistical analyses, confirm that the following items are present in the figure legend, table legend, main text, or Methods section.

n/a	Confirmed	
☐	☒	The exact sample size (n) for each experimental group/condition, given as a discrete number and unit of measurement
☐	☒	A statement on whether measurements were taken from distinct samples or whether the same sample was measured repeatedly
☐	☒	The statistical test(s) used AND whether they are one- or two-sided<br>*Only common tests should be described solely by name; describe more complex techniques in the Methods section.*
☒	☐	A description of all covariates tested
☐	☒	A description of any assumptions or corrections, such as tests of normality and adjustment for multiple comparisons
☐	☒	A full description of the statistical parameters including central tendency (e.g. means) or other basic estimates (e.g. regression coefficient) AND variation (e.g. standard deviation) or associated estimates of uncertainty (e.g. confidence intervals)
☐	☒	For null hypothesis testing, the test statistic (e.g. F, t, r) with confidence intervals, effect sizes, degrees of freedom and P value noted<br>*Give P values as exact values whenever suitable.*
☒	☐	For Bayesian analysis, information on the choice of priors and Markov chain Monte Carlo settings
☒	☐	For hierarchical and complex designs, identification of the appropriate level for tests and full reporting of outcomes
☐	☒	Estimates of effect sizes (e.g. Cohen's d, Pearson's r), indicating how they were calculated

Our web collection on statistics for biologists contains articles on many of the points above.

Software and code

Policy information about availability of computer code

Data collection	NA
Data analysis	Software used:<br>FASTQC (v0.11.5)<br>Timmomatic (v0.32)<br>Bowtie2 (v2.3.1)<br>Bowtie (v1.2.2)<br>Samtools (v1.7)<br>MACS2 (v2.1.2)<br>deepTools (v3.1.2)<br>Homer (v4.1)<br>STAR (v2.7.3)<br>DeSeq2 (v3.9)<br>DANPOS (v3.0)<br>BEDTools (v2.26.0)<br>AgriGO (v2.0)<br>PRISM (v8.0)<br>PyMOL (v2.3)<br>Code:<br>https://github.com/sklasfeld/ChIP_Annotation

For manuscripts utilizing custom algorithms or software that are central to the research but not yet described in published literature, software must be made available to editors/reviewers. We strongly encourage code deposition in a community repository (e.g. GitHub). See the Nature Research guidelines for submitting code & software for further information.

Data

Policy information about availability of data

All manuscripts must include a data availability statement. This statement should provide the following information, where applicable:

- Accession codes, unique identifiers, or web links for publicly available datasets
- A list of figures that have associated raw data
- A description of any restrictions on data availability

The authors declare that the data supporting the findings of this study are available within the paper and its supplementary information files. Source data are provided with this paper. The ChIP-seq, MNase-seq and RNA-seq datasets generated in this study are available at the GEO repository under accession number GSE141706.

Field-specific reporting

Please select the one below that is the best fit for your research. If you are not sure, read the appropriate sections before making your selection.

[x] Life sciences [ ] Behavioural & social sciences [ ] Ecological, evolutionary & environmental sciences

For a reference copy of the document with all sections, see nature.com/documents/nr-reporting-summary-flat.pdf

Life sciences study design

All studies must disclose on these points even when the disclosure is negative.

Sample size	The sample size was chosen on the basis of prior studies that showed significant effects with similar samples sizes (for example see Winter et al., Developmental Cell 2011, Xiao et al., Nature Genetics 2017 and Yamaguchi N. et al., Science 2015, Chung et al., NC 2020)
Data exclusions	No data was excluded from the analysis
Replication	All attempts at replication were sucessfull
Randomization	Sample allocation into groups was random
Blinding	The investigators were not blinded

Reporting for specific materials, systems and methods

We require information from authors about some types of materials, experimental systems and methods used in many studies. Here, indicate whether each material, system or method listed is relevant to your study. If you are not sure if a list item applies to your research, read the appropriate section before selecting a response.

Materials & experimental systems

n/a	Involved in the study	
[ ]	[x]	Antibodies
[x]	[ ]	Eukaryotic cell lines
[x]	[ ]	Palaeontology
[x]	[ ]	Animals and other organisms
[x]	[ ]	Human research participants
[x]	[ ]	Clinical data

Methods

n/a	Involved in the study	
[ ]	[x]	ChIP-seq
[x]	[ ]	Flow cytometry
[x]	[ ]	MRI-based neuroimaging

Antibodies

Antibodies used	rabbit polyclonal anti-LFY antibody (1:100 dilution)<br>Mouse IgG2bκ Anti-HA antibody (Abcam, 46540; 1:300 dilution)<br>Mouse anti-HA antibody (ROCHE, 12CA5; 1:300 dilution)<br>Rabbit Anti-Histone H3 antibody (Abcam, 18521; 1:300 dilution)<br>Rabbit Anti-Histone H1 antibody (Abcam, 61177; 1:300 dilution)
Validation	The commerical antibodies were validated by the suppliers. The LFY antibody was validated in Wagner et al. Science 1999

ChIP-seq

Data deposition

☒ Confirm that both raw and final processed data have been deposited in a public database such as GEO.

☒ Confirm that you have deposited or provided access to graph files (e.g. BED files) for the called peaks.

Data access links
May remain private before publication.

For "Initial submission" or "Revised version" documents, provide reviewer access links. For your "Final submission" document, provide a link to the deposited data.

Files in database submission

Deposition at GEO: GSE141706
ChIPseq
LFY ChIP mock treated: 3 replicates
LFY ChIP dex treated: 3 replicates
LFY ChIP input 3 replicates

RNA-seq
1hr Mock treated: 2 replicates
1hr Dex treated: 2 replicates
6hr Mock treated: 2 replicates
6hr Dex treated: 2 replicates
24hr Mock treated: 2 replicates
24hr Dex treated: 2 replicates

MNase-seq
Low digestion LFY-GR mock treated: 2 replicates
Low digestion LFY_GR dex treated: 2 replicates
High digestion LFY-GR mock treated: 2 replicates
High digestion LFY-GR dex treated: 2 replicates

Genome browser session
(e.g. UCSC)

Provide a link to an anonymized genome browser session for "Initial submission" and "Revised version" documents only, to enable peer review. Write "no longer applicable" for "Final submission" documents.

Methodology

Replicates

Replicates agreed well, data provided in the supplement

Sequencing depth

Experiment	Totalnumber of reads	Reads with MAPQ>=30	Length of reads	Paired or single end
Input Rep1	26,761,827	6,301,384	75	SINGLE
Input Rep2	20,718,056	5,069,260	75	SINGLE
Input Rep3	24,978,497	5,976,131	75	SINGLE
mock LFY ChIP Rep1	11,723,349	15,855,482	75	SINGLE
mock LFY ChIP Rep2	10,859,703	11,254,214	75	SINGLE
mock LFY ChIP Rep3	12,077,242	13,627,606	75	SINGLE
dex LFY ChIP Rep1	14,275,060	3,592,525	75	SINGLE
dex LFY ChIP Rep2	13,160,663	5,057,687	75	SINGLE
dex LFY ChIP Rep3	13,724,721	4,987,344	75	SINGLE

Antibodies

Antibody was validated Wagner et al. Science 1999 and by control ChIP reactions using plants lacking the antigene. data presented in the Supplement

Peak calling parameters

Peak calling is described in the Methods

Data quality

Peak attributes are described in the Methods

Software

Software used is described in the Methods
